# Do Causes Influence Functional Aspects and Quality of Life in Patients with Nonfibrocystic Bronchiectasis?

**DOI:** 10.1155/2024/3446536

**Published:** 2024-04-15

**Authors:** Ádria Cristina da Silva, Jessica de Campos Medeiros, Monica Corso Pereira

**Affiliations:** ^1^School of Medical Sciences, University of Campinas (UNICAMP), Campinas, SP, Brazil; ^2^Department of Internal Medicine, School of Medical Sciences, University of Campinas (UNICAMP), Campinas, SP, Brazil

## Abstract

**Background:**

The denomination of noncystic fibrosis bronchiectasis (NCFB) includes several causes, and differences may be expected between the patient subgroups regarding age, comorbidities, and clinical and functional evolution. This study sought to identify the main causes of NCFB in a cohort of stable adult patients and to investigate whether such conditions would be different in their clinical, functional, and quality of life aspects.

**Methods:**

Between 2017 and 2019, all active patients with NCFB were prospectively evaluated searching for clinical data, past medical history, dyspnea severity grading, quality of life data, microbiological profile, and lung function (spirometry and six-minute walk test).

**Results:**

There was a female predominance; mean age was 54.7 years. Causes were identified in 82% of the patients, the most frequent being postinfections (*n* = 39), ciliary dyskinesia (CD) (*n* = 32), and chronic obstructive pulmonary disease (COPD) (*n* = 29). COPD patients were older, more often smokers (or former smokers) and with more comorbidities; they also had worse lung function (spirometry and oxygenation) and showed worse performance in the six-minute walk test (6MWT) (walked distance and exercise-induced hypoxemia). Considering the degree of dyspnea, in the more symptomatic group, patients had higher scores in the three domains and total score in SGRQ, besides having more exacerbations and more patients in home oxygen therapy.

**Conclusions:**

Causes most identified were postinfections, CD, and COPD. Patients with COPD are older and have worse pulmonary function and more comorbidities. The most symptomatic patients are clinically and functionally more severe, besides having worse quality of life.

## 1. Introduction

Patients with bronchiectasis present recurrent respiratory infections that are both a cause and consequence of chronic inflammation in the airways. Over time, patients may present functional status decline and impairment of quality of life [1, 2]. In Brazil, data from the Ministry of Health identified 0.9 hospitalizations for bronchiectasis per 100,000 inhabitants and a mortality rate of 0.2/100,000 inhabitants [3].

Bronchiectasis is diagnosed by high-resolution computed tomography (HRCT) and may be associated with several etiologies [1, 2]. After diagnosis, it is important to try to identify an etiology, as some conditions may benefit from specific therapy [4].

The grouping currently called bronchiectasis not associated with cystic fibrosis (NCFB) comprises clinically and functionally heterogeneous patients. This classification brings together different causes and diseases into a single grouping, and clinical and pulmonary function differences between some conditions associated with NCFB have been described in literature [5–9]. There may be data from the patient's past history, as well as the age group affected or comorbidities that favor a specific cause. In addition, it is relevant to expand the assessment of the functional impact and quality of life in different groups, in order to recognize their particularities. The recognition of these clinical and functional differences can help to improve and personalize the clinical and therapeutic management of these patients.

This study sought to identify the main causes of NCFB in a cohort of stable adult patients and to investigate whether such conditions would be different in their clinical, functional, and quality of life aspects.

## 2. Methods

We recruited patients diagnosed with bronchiectasis by HRCT from the medical records (radiological reports). The etiology was identified by reviewing the medical files. Patients with cystic fibrosis were not included.

Between 2017 and 2019, all active patients aged 18 years or higher with NCFB were included. All participants were interviewed for collecting clinical data, past medical history, and dyspnea severity grading (mMRC) and answered a quality of life assessment questionnaire. The microbiological data (sputum sample) was analyzed. All patients included were invited to perform functional tests (spirometry and walking test), and those who accepted and had appropriate physical conditions underwent the tests. The study was approved by the local Research Ethics Committee (report number 69779517.4.0000.5404), and written informed consent was obtained from each patient.

The postinfectious cause was defined when the patient reported symptom onset or recurrent pneumonia after a severe infectious event, or when the patient was treated for tuberculosis (TB) and showed consistent findings on HRCT. The diagnosis of chronic obstructive pulmonary disease (COPD) was established according to the GOLD criteria [8] in patients with a smoking history of more than 10 pack-years. Ciliary dyskinesia (CD) was defined by the presence of diffuse bronchiectasis associated with *situs inversus* (Kartagener syndrome), or with infertility and chronic sinusitis. Immunodeficiency is defined by evidence of permanently reduced immunoglobulins (IgM and IgG). Other causes were confirmed or ruled out as appropriate. At our institution, all patients with diffuse bronchiectasis underwent a sweat chloride test to rule out the diagnosis of CF.

Clinical data and past medical history were collected using a data collection form.

We asked participants about several risk factors, including those inherent to the subject (delivery conditions, low birth weight), as well as those acquired throughout life, such as childhood infections (measles, pneumonia, and pertussis), previous treatment for tuberculosis, exposure to passive or active smoking, exposure to wood stove smoke, previous diagnosis of asthma, and comorbidities. The patients answered the St. George's Respiratory Questionnaire (SGRQ), which comprises three domains: symptoms, activity, and psychosocial impact of respiratory diseases. The higher the value obtained, the worse the quality of life [9].

The 6MWT was performed under the supervision of the same technician, following the American Thoracic Society guidelines [10]. We collected the distance covered (meters) and calculated its correlation with values predicted by international [11] and national [12, 13] reference equations. Oxygen desaturation was defined by a drop in saturation of more than 4% between the first and sixth minutes, or when the final SpO_2_ was less than 89%. Spirometry was performed under the recommendations of the Brazilian Thoracic Society, and Brazilian reference values were used [12]. Spontaneous sputum samples were collected for microbiological analysis.

A senior pulmonologist (MCP) with experience in reading CT scans reviewed all tomographic images available to identify the number of lung lobes involved and the presence of emphysema or air trapping. The severity of bronchiectasis was assessed using the FACED prognostic score [14].

### 2.1. Statistical Analysis

Data were presented either as mean ± standard deviation (±SD) or frequency measures (categorical variables). For comparison between causes, the Kruskal-Wallis test was used, followed by Dunn's test (quantitative variables) and chi-square or Fisher's exact test. Univariate and multivariate logistic regression analyses were used to identify associations between the outcomes of interest. Results of univariate and multivariate analyses were generated as odds ratios (ORs) and 95% confidence intervals (CIs). Oligosymptomatic patients were compared with more symptomatic patients (mMRC scale) using the Mann–Whitney test, chi-square, or Fisher's exact test. A *p* value < 0.05 was considered significant for all statistical analyses. All calculations were performed with the SAS for Windows (Statistical Analysis System, version 9.4, SAS Institute Inc., 2002-2012, Cary, NC, USA; R version 3.4.2, Copyright © 2017 The R Foundation for Statistical Computing).

## 3. Results

We recruited a total of 384 patients with bronchiectasis, of which 217 were not included (110 due to follow-up for less than six months at the institution, 88 diagnosed with CF, and 19 deaths). Thus, 167 patients had their medical records reviewed to establish the causes, and 126 were interviewed. Not all of them accepted or had the appropriate physical conditions, so the number of patients who underwent the 6MWT is smaller than the total number of the sample (*N* = 84). No deaths were observed during the period examined.

Of the 167 patients, there was a female predominance, the mean age was 54.7 years, and the mean BMI was 25.5 kg/m^2^. The most reported symptoms were wheezing (68%) and productive cough (55.9%). Causes were identified in 82% of the patients, the most frequent being postinfections (*n* = 39), followed by CD (*n* = 32), and COPD (*n* = 29). Hypertension was the most frequently found comorbidity (43.4%).  Table 1 shows the data of all patients.

A comparative analysis was performed between the most frequently identified causes of bronchiectasis (Table 2). Twenty-four patients out of 167 had other causes, such as bronchiectasis after bone marrow transplantation, allergic bronchopulmonary aspergillosis, rheumatoid arthritis, asthma, alpha 1 antitrypsin deficiency, mixed connective tissue disease, ulcerative colitis, and Young's syndrome. As there were few patients in each of these other causes, these 24 patients were not included in the comparison between groups.

COPD patients had higher age, higher smoking frequency, higher smoking load, and more comorbidities by Charlson's comorbidity index (CCI) [15]. Functionally, COPD patients had lower SpO_2_ and worse lung function; on 6MWT, they walked shorter distances and had more frequent desaturation. For comparison of distances, there was no difference between groups using international [11] or Brazilian [12, 13] reference equations.

At least one positive culture for *Pseudomonas aeruginosa* was identified in 54.3% of patients, and 38.6% had two or more positive cultures. All groups had isolates positive for *P. aeruginosa*, with no difference between groups in the prevalence of *P. aeruginosa* or other identified microorganisms. There was also no difference between the groups regarding respiratory symptoms and SGRQ scores.

Univariate and multivariate logistic regression models were used to examine the relationship between patients with chronic bronchial infection with demographic, clinical, and functional characteristics. Univariate logistic regression analysis indicated that advanced age, worse lung function, immunomodulatory therapy (use of macrolides), and home oxygen therapy increased the chances of chronic bronchial infection. In the multivariate analysis, only patients using home oxygen therapy (OR, 7,250; CI 1,210-43,442; *p* = 0.0301) were 7.2 times more likely to develop chronic bronchial infection.

Considering the severity of dyspnea, we compared 89 fewer symptomatic patients (mMRC 0-1) with 78 more symptomatic patients (mMRC 2 or more) (Table 3). There was a statistically significant difference in spirometry (FVC and FEV1), baseline SpO_2_, and 6MWT walking distance. In the symptomatic group, women predominated with higher scores in all three domains and total SGRQ score, as well as having more exacerbations and more patients on home oxygen therapy.

## 4. Discussion

In this study, we identified at least one cause in 82% of cases, a finding similar to other studies [4, 16]. The frequency of unidentified causes for NCFB ranges from 20 to 46% [4, 6, 16], and this large variation may result from several factors, such as different research protocols or inclusion criteria applied, the prevalence of infectious diseases, socioeconomic status of the study population, access to healthcare treatment, and vaccination coverage. There is a difference in epidemiology between developed and underdeveloped countries [17]. A large review evaluated the causes among adults and identified that idiopathic bronchiectasis is more frequently found in Asia and Oceania when compared with Europe, South America, Africa, and North America [18]. The postinfectious cause was the most prevalent, also similar to other studies [4, 16].

In our sample, the mean age of 54.7 years and the predominance of women comprise a profile similar to that of other studies [4, 6, 19]. Martínez-García et al. [14] found a mean age of 58.7 years, and in the Latin American validation of the FACED score [19], the mean age was 48.2 years.

COPD was the third most commonly identified cause, with a frequency similar to that of the Lonni et al. study [6]. In contrast to our study, in a multicenter study of 2.195 patients, mostly men and without a history of smoking, COPD was a less common cause [20]. On the other hand, the study of patients in advanced stages of COPD detects a high prevalence of bronchiectasis [21].

Compared with other causes, COPD patients were older and had worse lung function, lower SpO_2_ on room air, and more comorbidities assessed both individually and by CCI [15]. There is evidence that patients with COPD-associated bronchiectasis have more severe airway obstruction, as well as a higher risk of exacerbations, longer medical follow-up, higher rates of hospitalization, and higher mortality [22]. In an analysis of six European databases [23], CCI > 1 was associated with older patients and worse outcomes. McDonnell et al. [24] identified more comorbidities in males with COPD, in addition to the association with worse survival, suggesting that these patients would have a worse prognosis when compared with other causes.

The mean total SGRQ score indicated moderate impairment to quality of life, and the activity domain showed higher scores, followed by the domain symptoms and psychosocial impact. Similar data found in another study [25] reinforce the impact of bronchiectasis disease on the individual's life. No difference was found between the groups regarding the quality of life.

The mean distance walked was 460.5 meters, and although COPD patients walked shorter distances (in meters), there was no statistical difference between the groups in the comparison using the various reference equations. The distance walked varies among the populations studied, reflecting demographic characteristics and the diverse physical abilities among populations. Some authors show values close to those found in our study [26, 27], while others show longer walking distances [28]. It should be emphasized that in these studies with a longer average distance, patients with COPD were not included, and the evaluated population had lower severity.

Another relevant result was that 29.9% of patients had exercise-induced desaturation, and this was more frequent in patients with COPD. The criteria used here to define desaturation showed good reproducibility for use in clinical practice [29]. This seems to be a frequent finding in COPD patients [30] and associated with a worse prognosis [31]. The presence of desaturation is also associated with more symptoms [27], shorter walking distances, and worse lung function [27, 29].

Comparing patients according to the degree of dyspnea, more symptomatic patients had a higher frequency of exacerbations and hospitalizations, more frequent use of home oxygen therapy, lower SpO_2_ on room air, lower variables on spirometry and 6MWT, and, in addition, worse scores in all domains of the SGRQ. These findings are similar to Nucci et al. [32] and reinforce the importance of this simple measure to assess the severity of the bronchiectasis patient. As a heterogeneous disease, there is no single variable capable of capturing the severity of NCFB patients. In this context, some multidimensional scores have been developed and validated, such as FACED [14] and BSI [33]. Both scores comprise the assessment of dyspnea; however, it is important to recognize the strength of this measure alone, a fact corroborated in this study.

A limitation of our study is the retrospective nature of data collection to identify causes, as this may result in missing data. Personal antecedents were collected through interviews, and some data, especially referring to birth and the perinatal period, may not be clearly remembered (memory bias). Despite this, we consider this to be relevant information, given the scarcity of data regarding morbid and childhood antecedents in studies with patients diagnosed with bronchiectasis. Another limitation that can be pointed out is that the CT scans were evaluated by only one senior and experienced pulmonologist with CT scan reading, and not by two or more radiologists, in order to avoid bias and misinterpretation of the findings. However, this was not possible to accomplish. Considering that the reading was done systematically and focused only on grading the extent of structural alterations, we consider that this fact does not significantly impact the data presented here.

Despite coming from a unicentric study, the data presented here, due to their completeness, contribute to understanding the heterogeneity of patients who have been designated as having noncystic fibrosis bronchiectasis.

## 5. Conclusions

Even with systematization in the investigation of causes of bronchiectasis, almost one-fifth of the patients evaluated here remained with an undefined cause. However, it was possible to identify potentially treatable causes such as immunodeficiencies (9%) and ciliary dyskinesia (22%). These findings reinforce current recommendations regarding the minimal etiological investigation of bronchiectasis [1, 34].

The main causes of bronchiectasis identified were postinfections, CD, and COPD. Comparing the identified causes, patients with COPD are older, have worse lung function and lower SpO_2_, have more comorbidities, and walked shorter distances in the 6MWT. The most symptomatic patients are clinically and functionally more severe and have a worse quality of life.

It should also be noted the high prevalence of chronic infection by *P. aeruginosa* in patients with bronchiectasis: of the analyzed sample, 38.6% had two or more positive cultures for *P. aeruginosa*. The prevalence of chronic *P. aeruginosa* infection was high in all groups separated by causes (from 20 to 31%) with no difference between them. This finding is known to require specific management because it is associated with greater functional decline and more frequent exacerbations. Thus, every patient diagnosed with bronchiectasis must be actively screened for persistence of pathogenic microorganisms in airway secretions.

## Figures and Tables

**Table 1:** Baseline clinical, etiologic, functional, and quality of life data of patients with bronchiectasis.

Variables	*N* = 167
*Clinical and etiologic data*	
Sex	
Female	96 (57)
Male	71 (43)
Age (years)	54.7 ± 16.0
BMI (kg/m^2^)	25.5 ± 5.8
Symptoms	
Dry cough, yes	34 (26.6)
Productive cough, yes	71 (55.9)
Wheezing, yes	87 (68.0)
Smoking history, yes	
Former smokers, yes	55 (32.9)
Current smokers, yes	6 (3.6)
Wood stove exposure, yes	67 (45.0)
Causes	
Postinfectious	39 (23.4)
Ciliary dyskinesia	32 (19.2)
COPD	29 (17.4)
Immunodeficiency	13 (7.8)
Idiopathic	30 (18.0)
Other causes^∗^	24 (14.4)
No. of exacerbations, mean per year (*n* = 155)	1.1 ± 1.5
Comorbidities, *n* (%)	
Systemic arterial hypertension (SAH), yes	72 (43.4)
Diabetes mellitus, yes	34 (20.7)
Dyslipidemia, yes	26 (15.8)
Charlson's comorbidity index (CCI)	2.7 ± 2.1
Drug treatment	
ICS	116 (73.0)
Systemic corticosteroid	11 (6.9)
LABA	43 (27.0)
SABA	19 (11.9)
LAMA	67 (42.1)
SAMA	9 (5.7)
Macrolide	58 (36.5)
Home oxygen therapy, yes	24 (14.8)
*Tomographic findings (n* = 148*)^#^*	
No. of pulmonary lobes affected, *n* (%)	
1 to 3 lobes	38 (25.7)
4 to 6 lobes	110 (74.3)
Emphysema	15 (10.1)
Air trapping (*n* = 123)^∗∗^	102 (82.9)
FACED score (*n* = 148)	
Light (0-2 points)	70 (47.3)
Moderate (3-4 points)	55 (37.2)
Severe (5-7 points)	23 (15.5)
Functional data	
SpO_2_ (%)	95.1 ± 3.8
Lung function (*n* = 159)	
FVC (L)	2.3 ± 0.9
FVC (% of predicted value)	64.4 ± 17.5
FEV1 (L)	1.6 ± 0.8
FEV1 (% of predicted value)	54.8 ± 22.1
FEV1/FVC	0.7 ± 0.1
Obstructive ventilatory disturb, yes	6 (3.8)
Obstructive ventilatory disturb with reduced FVC, yes	30 (18.9)
Preserved ratio impaired spirometry, yes	66 (41.5)
Restrictive ventilatory disturb, yes	14 (8.8)
6MWT (*n* = 84)	
Walking distance (6MWD) (m)	460.5 ± 102.2
6MWD as % of predicted value according to different prediction equations	
Enright and Sherril [11]	78.2 ± 15.4
Iwama et al. [12]	82.4 ± 16.6
Soares and Pereira [13]	85.5 ± 15.2
Saint George's Hospital Questionnaire (SGRQ)	
Symptom	45.0 ± 26.2
Activity	59.3 ± 29.2
Psychosocial impact	35.2 ± 22.7
Total	45.4 ± 23.5

Values expressed as mean ± standard deviation or as measures of frequency, as appropriate. ^∗^See details in the text. ^∗∗^Air trapping was analyzed only in scans with an expiratory phase. ^#^In 19 patients, the images could not be recovered for prospective analysis. BMI: body mass index; CCI: Charlson's comorbidity index (0-37); ICS: inhaled corticosteroid; LABA: long-acting beta agonist; SABA: short-acting beta agonist; LAMA: long-acting muscarinic antagonist; SAMA: short-acting muscarinic antagonist; SpO_2_: peripheral oxygen saturation; FVC: forced vital capacity; FEV1: forced expiratory volume in the first second; SGRQ: Saint George's Hospital Questionnaire, scores presented as mean and SD (range 0-100).

**Table 2:** Comparison between the causes of bronchiectasis (*N* = 143^#^).

Variables	Idiopathic (*n* = 30)	Postinfectious (*n* = 39)	CD (*n* = 32)	COPD (*n* = 29)	Immunodeficiencies (*n* = 13)	*p* value
*Clinical data*						
Age (years)	61.9 ± 11.9	54.6 ± 13.8	42.9 ± 16.1	66.8 ± 12.2	47.3 ± 16.2	<0.0001
Female gender, *n* (%)	18 (60.0)	28 (71.8)	12 (37.5)	13 (44.8)	8 (61.5)	0.038
BMI (kg/m^2^)^∗^	25.7 ± 7.0	26.6 ± 4.8	24.2 ± 4.8	25.7 ± 6.9	23.8 ± 5.1	0.276
Symptoms, *n* (%)						
Cough	19 (82.6)	23 (76.7)	20 (90.9)	15 (71.4)	10 (90.9)	0.479
Dry cough	6 (26.1)	7 (23.3)	5 (22.7)	5 (23.8)	2 (18.2)	0.992
Productive cough	13 (56.5)	16 (55.2)	15 (68.2)	10 (47.6)	8 (72.7)	0.565
Wheezing	15 (65.2)	22 (73.3)	17 (77.3)	12 (57.1)	6 (54.5)	0.504
Dyspnea (mMRC)						
0-1	15 (50.0)	16 (41.0)	23 (71.9)	12 (41.4)	8 (61.5)	0.067
≥2	15 (50.0)	23 (59.0)	9 (28.1)	17 (58.6)	5 (38.5)	
Risk factors						
Tobacco exposure, *n* (%)						
Smoker	7 (23.3)	16 (41.0)	6 (18.8)	20 (69.0)	4 (30.8)	0.001
Pack-years	14.0 ± 32.0	14.5 ± 20.6	2.9 ± 6.8	34.8 ± 37.1	5.8 ± 11.1	<0.01
Maternal smoking	12 (42.9)	8 (2)	3 (17.7)	9 (56.3)	1 (12.5)	0.090
Paternal smoking	16 (57.1)	21 (84.0)	11 (64.7)	9 (56.3)	5 (62.5)	0.256
Smoking mother during pregnancy	7 (25.0)	4 (16.0)	0 (0.0)	5 (31.3)	1 (12.5)	0.105
Wood stove exposure (years), *n* (%)	13.8 ± 14.4	13.7 ± 17.5	10.9 ± 16.2	13.4 ± 12.2	8.1 ± 11.4	0.696
Personal and familial antecedents, *n* (%)						
Preterm birth	2 (7.1)	4 (16.0)	4 (23.5)	2 (12.5)	0 (0.0)	0.475
RNF	6 (21.4)	3 (12.0)	5 (29.4)	1 (6.3)	0 (0.0)	0.268
Pneumonia	14 (50.0)	9 (36.0)	10 (58.8)	2 (12.5)	1 (12.5)	0.029
Pertussis	6 (21.4)	8 (32.0)	2 (11.8)	3 (18.8)	0 (0.0)	0.342
Tuberculosis, yes (*n* = 124)	3 (11.5)	23 (65.7)	5 (17.9)	1 (4.3)	1 (8.3)	<0.0001
Measles	16 (57.1)	14 (56.0)	8 (47.1)	9 (56.3)	2 (25.0)	0.545
Otitis	6 (21.4)	5 (20.0)	6 (35.3)	3 (18.8)	3 (37.5)	0.649
Sinusitis	11 (39.3)	7 (28.0)	7 (41.2)	5 (31.3)	6 (75.0)	0.193
Exacerbations (last year)	1.6 ± 1.8	1.1 ± 1.6	0.8 ± 1.4	1.2 ± 1.5	1.4 ± 1.6	0.229
Hospitalization (last year)	0.3 ± 0.7	0.2 ± 0.4	0.1 ± 0.3	0.5 ± 0.8	0.1 ± 0.3	0.103
Hospital days	17.0 ± 21.3	9.7 ± 4.9	5.3 ± 2.9	9.2 ± 7.7	10.0 ± 0	0.528
Comorbidities, *n* (%)						
Systemic arterial hypertension	17 (56.7)	17 (43.6)	6 (18.8)	18 (62.1)	1 (8.3)	0.001
Diabetes mellitus	9 (30.0)	8 (20.5)	1 (3.1)	8 (28.6)	1 (8.3)	0.038
Dyslipidemia	6 (20.7)	7 (17.9)	0 (0.0)	9 (31.0)	1 (8.3)	0.006
CCI	2.8 ± 1.8	2.4 ± 1.7	1.6 ± 1.7	4.7 ± 2.5	1.9 ± 1.0	<0.0001
Drug treatment, *n* (%)						
ICS	22 (78.6)	23 (60.5)	23 (74.2)	23 (79.3)	9 (81.8)	0.345
Systemic corticosteroid	1 (3.6)	2 (5.3)	2 (6.5)	3 (10.3)	0 (0.0)	0.84
LABA	8 (28.6)	12 (31.6)	9 (29.0)	8 (27.6)	3 (27.3)	0.997
SABA	2 (7.1)	8 (21.1)	3 (9.7)	5 (17.2)	0 (0.0)	0.308
LAMA	11 (39.3)	17 (44.7)	11 (35.5)	21 (72.4)	3 (27.3)	0.021
SAMA	0 (0.0)	4 (10.5)	2 (6.5)	2 (6.9)	1 (9.1)	0.455
Macrolide	12 (42.9)	11 (28.9)	12 (38.7)	10 (34.5)	7 (63.6)	0.305
Home oxygen therapy	4 (13.3)	6 (16.7)	3 (9.4)	7 (25.0)	1 (8.3)	0.546
*Functional data*						
SpO_2_ (%)	94.0 ± 5.5	96.1 ± 2.2	96.0 ± 2.6	93.6 ± 3.7	94.8 ± 5.0	0.026
FVC (L)	2.0 ± 0.7	2.1 ± 0.8	2.8 ± 1.0	2.0 ± 0.7	2.9 ± 1.1	0.001
FVC (% of predicted)	63.0 ± 18.7	63.2 ± 16.0	66.6 ± 20.1	59.1 ± 12.9	75.1 ± 17.9	0.146
FEV_1_ (L)	1.4 ± 0.6	1.5 ± 0.7	2.0 ± 0.9	1.1 ± 0.5	2.3 ± 1.0	<0.01
FEV_1_ (% of predicted)	56.1 ± 20.8	53.1 ± 20.7	57.5 ± 25.0	42.3 ± 15.4	69.7 ± 23.8	0.006
FEV_1_/FVC	0.7 ± 0.1	0.7 ± 0.1	0.7 ± 0.2	0.6 ± 0.1	0.8 ± 0.1	<0.01
6MWT (*n* = 67)						
6MWD (m)	439 ± 122	445 ± 105.1	505 ± 94.4	392 ± 117.5	514 ± 19.4	0.008
6MWD as % of predicted value according to different prediction equations						
Enright and Sherrill [11]^∗^	79.7 ± 14.3	78.2 ± 12.8	78.6 ± 14.9	79.2 ± 22.8	78.3 ± 16.0	0.988
Iwama et al. [12]^∗^	81.1 ± 19.4	81.2 ± 16.6	87.1 ± 15.8	71.9 ± 21.6	87.9 ± 4.3	0.195
Soares and Pereira [13]^∗^	85.2 ± 17.6	83.2 ± 15.1	87.5 ± 13.1	81.8 ± 23.6	87.3 ± 7.7	0.940
Desaturation^∗∗^	3 (23.1)	1 (7.7)	4 (18.2)	9 (69.2)	3 (50.0)	0.004
*Microbiological culture of sputum*						
*P. aeruginosa* (ever)	10 (52.6)	11 (42.3)	7 (28.0)	7 (36.8)	3 (30.0)	0.516
*P. aeruginosa* (≥2 last year)	6 (31.6)	8 (30.8)	6 (25.0)	5 (26.3)	2 (20.0)	0.955
*Quality of life (SGRQ)* (*n* = 126)						
Symptoms	46.1 ± 27.7	49.4 ± 25.5	49.5 ± 28.2	39.5 ± 26.1	45.2 ± 28.6	0.724
Activity	58.0 ± 29.8	68.1 ± 28.9	47.5 ± 27.9	68.4 ± 28.1	52.7 ± 36.3	0.069
Psychosocial impact	36.5 ± 24.2	42.4 ± 20.0	28.1 ± 24.3	38.0 ± 22.1	37.7 ± 24.3	0.184
Total	45.8 ± 24.8	53.0 ± 21.1	38.5 ± 25.0	49.0 ± 23.7	44.8 ± 26.4	0.267

Values expressed as mean ± standard deviation or as measures of frequency, as appropriate. ^#^24 patients out of 167 have multiple causes and were excluded of this comparative analysis (see text for more details). ^∗^% of predicted value according to different prediction equations. ^∗∗^No. of patients who presented SpO_2_ drop at the end of the 6MWT ≤ 88%, or 4-point drop of initial SpO_2_. BMI: body mass index; RNF: neonatal respiratory failure; CCI: Charlson's comorbidity index; ICS: inhaled corticosteroid; LABA: long-acting beta agonist; SABA: short-acting beta agonist; LAMA: long-acting muscarinic antagonist; SAMA: short-acting muscarinic antagonist; FVC: forced vital capacity; FEV1: forced expiratory volume in the first second; SpO_2_: peripheral oxygen saturation; mMRC: Medical Research Council; 6MWT: six-minute walk test; COPD: chronic obstructive pulmonary disease; CD: ciliary dyskinesia; SGRQ: Saint George's Hospital Questionnaire on respiratory disease.

**Table 3:** Comparison of patients according to the degree of dyspnea: clinical, functional, and quality of life (*n* = 167).

Variables	Few symptoms (mMRC 0-1) (*n* = 89)	Very symptomatic (mMRC ≥ 2) (*n* = 78)	*p* value
*Clinical data*			
Age (years)	52.2 ± 16.4	57.4 ± 15.1	0.088
Female gender, *n* (%)	44 (49.4)	52 (66.7)	0.025
Male gender, *n* (%)	45 (50.6)	26 (33.3)	
BMI (kg/m^2^)	24.8 ± 5.7	26.3 ± 5.9	0.232
Symptoms, *n* (%)			
Cough	53 (79.1)	52 (85.2)	0.366
Dry cough	20 (29.9)	14 (23.0)	0.377
Productive cough	33 (49.3)	38 (63.3)	0.111
Wheezing	41 (61.2)	46 (75.4)	0.085
Smoking, *n* (%)	26 (2.2)	35 (44.9)	0.036
Pack-years	8.9 ± 14.9	21.5 ± 34.4	0.062
Wood stove exposition, *n* (%)	36 (44.4)	31 (45.6)	0.889
Hospitalization (last year), *n* (%)	9 (11.0)	22 (30.1)	0.003
Exacerbation (last year), *n* (%)	33 (40.2)	46 (65.8)	0.002
Comorbidities, *n* (%)			
Systemic arterial hypertension	36 (40.9)	36 (46.2)	0.496
Diabetes mellitus	19 (21.8)	15 (19.5)	0.71
Dyslipidemia	15 (17.0)	11 (14.3)	0.627
CCI	2.6 ± 2.1	2.8 ± 2.1	0.372
Medications, *n* (%)			
ICS	60 (72.3)	56 (73.7)	0.843
Systemic corticosteroid	4 (4.8)	7 (9.2)	0.276
LABA	22 (26.5)	21 (27.6)	0.873
SABA	4 (4.8)	15 (19.7)	0.004
LAMA	29 (34.9)	38 (50.0)	0.055
SAMA	2 (2.4)	7 (9.2)	0.088
Macrolide	25 (30.1)	33 (43.4)	0.082
Home oxygen therapy	5 (5.8)	19 (25.0)	0.001
*Functional data*			
SpO_2_	96.2 ± 3.0	94.9 ± 2.8	0.01
Spirometry			
FVC (L)	2.5 ± 0.9	2.0 ± 0.7	<0.0001
FVC (% of predicted)	67.9 ± 17.8	60.2 ± 16.3	0.007
FEV_1_ (L)	1.8 ± 0.8	1.3 ± 0.6	<0.01
FEV_1_ (% of predicted)	59.0 ± 22.9	49.8 ± 20.2	0.011
FEV_1_/FVC	0.7 ± 0.1	0.6 ± 0.1	0.055
6MWT			
6MWD (m)	491 ± 88.6	419 ± 105.6	0.002
6MWD as % of predicted value according to different prediction equations			
Enright and Sherrill [11]^∗^	79.9 ± 14.8	76.0 ± 16.1	0.286
Iwama et al. [12]^∗^	86.7 ± 13.7	76.6 ± 18.4	0.012
Soares and Pereira [13]^∗^	88.5 ± 12.3	80.8 ± 17.6	0.036
Desaturation^∗∗^	14 (29.2)	12 (33.3)	0.683
*Quality of life data*			
Symptom	32.1 ± 23.4	59.7 ± 21.0	<0.0001
Activity	41.8 ± 25.7	79.2 ± 18.3	<0.0001
Psychosocial impact	22.4 ± 18.4	49.7 ± 18.0	<0.0001
Total	30.8 ± 19.1	59.7 ± 21.0	<0.0001

Values expressed as mean ± standard deviation or as measures of frequency, as appropriate. ^∗^% of predicted value according to different prediction equations. ^∗∗^SpO_2_ at the end of the 6MWT ≤ 88%, or 4-point drop of initial SpO_2_. BMI: body mass index; FVC: forced vital capacity; FEV1: forced expiratory volume in the first second; SpO_2_: peripheral oxygen saturation; mMRC: Medical Research Council; SAH: systemic arterial hypertension; CCI: Charlson's comorbidity index; ICS: inhaled corticosteroid; LABA: long-acting beta agonist; SABA: short-acting beta agonist; LAMA: long-acting muscarinic antagonist; SAMA: short-acting muscarinic antagonist.

## Data Availability

No underlying data was collected or produced in this study.
